# Genetic Spectrum of Autosomal Recessive Non-Syndromic Hearing Loss in Pakistani Families

**DOI:** 10.1371/journal.pone.0100146

**Published:** 2014-06-20

**Authors:** Sobia Shafique, Saima Siddiqi, Margit Schraders, Jaap Oostrik, Humaira Ayub, Ammad Bilal, Muhammad Ajmal, Celia Zazo Seco, Tim M. Strom, Atika Mansoor, Kehkashan Mazhar, Syed Tahir A. Shah, Alamdar Hussain, Maleeha Azam, Hannie Kremer, Raheel Qamar

**Affiliations:** 1 COMSATS Institute of Information Technology, Park Road, Islamabad, Pakistan; 2 Institute of Biomedical and Genetic Engineering (IBGE), Islamabad, Pakistan; 3 Department of Otorhinolaryngology, Hearing and Genes, Radboud University Medical Center, Nijmegen, The Netherlands; 4 Nijmegen Centre for Molecular Life Sciences, Radboud University Medical Center, Nijmegen, The Netherlands; 5 Donders Institute for Brain, Cognition and Behaviour, Radboud University Medical Center, Nijmegen, The Netherlands; 6 Simon Fraser University, Vancouver, British Colombia, Canada; 7 Institute of Human Genetics, Helmholtz Zentrum München, German Research Center for Environmental Health, Neuherberg, Germany; 8 Department of Human Genetics, Radboud university medical center, Nijmegen, The Netherlands; 9 Al-Nafees Medical College & Hospital, Isra University, Islamabad, Pakistan; Instituto de Ciencia de Materiales de Madrid - Instituto de Biomedicina de Valencia, Spain

## Abstract

The frequency of inherited bilateral autosomal recessive non-syndromic hearing loss (ARNSHL) in Pakistan is 1.6/1000 individuals. More than 50% of the families carry mutations in *GJB2* while mutations in *MYO15A* account for about 5% of recessive deafness. In the present study a cohort of 30 ARNSHL families was initially screened for mutations in *GJB2* and *MYO15A*. Homozygosity mapping was performed by employing whole genome single nucleotide polymorphism (SNP) genotyping in the families that did not carry mutations in *GJB2* or *MYO15A*. Mutation analysis was performed for the known ARNSHL genes present in the homozygous regions to determine the causative mutations. This allowed the identification of a causative mutation in all the 30 families including 9 novel mutations, which were identified in 9 different families (*GJB2* (c.598G>A, p.Gly200Arg); *MYO15A* (c.9948G>A, p.Gln3316Gln; c.3866+1G>A; c.8767C>T, p.Arg2923* and c.8222T>C, p.Phe2741Ser), *TMC1* (c.362+18A>G), *BSND* (c.97G>C, p.Val33Leu), *TMPRSS3* (c.726C>G, p.Cys242Trp) and *MSRB3* (c.20T>G, p.Leu7Arg)). Furthermore, 12 recurrent mutations were detected in 21 other families. The 21 identified mutations included 10 (48%) missense changes, 4 (19%) nonsense mutations, 3 (14%) intronic mutations, 2 (9%) splice site mutations and 2 (9%) frameshift mutations. *GJB2* accounted for 53% of the families, while mutations in *MYO15A* were the second most frequent (13%) cause of ARNSHL in these 30 families. The identification of novel as well as recurrent mutations in the present study increases the spectrum of mutations in known deafness genes which could lead to the identification of novel founder mutations and population specific mutated deafness genes causative of ARNSHL. These results provide detailed genetic information that has potential diagnostic implication in the establishment of cost-efficient allele-specific analysis of frequently occurring variants in combination with other reported mutations in Pakistani populations.

## Introduction

Deafness or hearing loss is the most common congenital sensorineural disorder affecting about 1 in 1000 children. Genetic factors contribute to approximately half of the cases of hearing loss [1], which can be either syndromic or non-syndromic. The former is responsible for about 30% of prelingual deafness in combination with abnormalities of other organs. Non-syndromic deafness is usually due to abnormalities of the middle and/or the inner ear and is found in 70% of the hereditary cases [2]. The disease is genetically heterogeneous and currently 134 loci have been determined and 80 genes identified for the non-syndromic type (Hereditary Hearing Loss Homepage, URL: http://hereditaryhearingloss.org/). More than 400 syndromes are known with deafness as one of the symptoms and for many of these the causative genes have been identified (Online Mendelian Inheritance in Man: http://www.ncbi.nlm.nih.gov/omim).

Syndromic and non-syndromic hearing loss display an autosomal recessive, autosomal dominant, X-linked, Y-linked or mitochondrial pattern of inheritance [3]. Eighty percent of the early onset non-syndromic cases have an autosomal recessive inheritance pattern (ARNSHL), while autosomal dominant, X-linked and mitochondrial non-syndromic deafness contribute to 18%, 1–3% and <1% of the cases, respectively [4]. In Pakistan, hearing impairment is severe and congenital in 70% of the cases and the increased occurrence of these conditions is due to a high rate of consanguineous marriages (60%); profound bilateral deafness occurs at 1.6 per 1000 individuals [5].

The genes most frequently involved in ARNSHL are those encoding gap junction protein beta 2 (*GJB2*, MIM# 121011), myosin XVA (*MYO15A*, MIM# 602666), transmembrane channel-like 1 (*TMC1*, MIM# 606706), solute carrier family 26 (anion exchanger) member 4 (*SLC26A4*, MIM# 605646), otoferlin (*OTOF*, MIM# 603681) and cadherin-related 23 (*CDH23*, MIM# 605516), each of which has been found to contain more than 20 different mutations, most of which have been reported in consanguineous families [6]. Mutations in *GJB2* are the most common cause of ARNSHL and explain up to 50% cases in the Mediterranean regions [7], [8], [9] while mutations in multiple genes have been shown to cause deafness in the remaining cases. In the Pakistani population mutations in *MYO15A* account for 5% of the recessive deafness [10].

In the present study a panel of 30 unrelated consanguineous Pakistani families was initially tested for involvement of *GJB2* and *MYO15A* followed by whole genome homozygosity mapping and candidate gene sequencing. This approach resulted in defining the mutation spectrum of the disease in the current panel.

## Materials and Methods

### Ethics statement

The current study conformed to the tenets of the Helsinki declaration and was approved by the Department of Biosciences Ethics Review Board of the COMSATS Institute of Information Technology, Islamabad, Pakistan. All patients, their normal hearing family members and 89 ethnically matched control individuals were informed about the purpose of the study and written consent was taken before recruitment and sampling. Informed written consent of minors was obtained from their guardians.

### Genotyping

A total of 30 consanguineous families with ARNSHL were ascertained from different regions of Punjab, Pakistan. Audiometery was performed on a few members from each family to determine the level of hearing loss.

Blood samples were collected in EDTA containing vacutainers. DNA extraction from these samples was carried out using a standard phenol-chloroform/organic method [11]. Microsatellite markers were analyzed as described previously [12] and Sanger sequencing was performed according to Schraders et al. [13]. Primers were designed using Primer3 software (URL: http://www.bioinformatics.nl/cgi-bin/primer3plus/primer3plus.cgi/; [14]). Primer sequences are available upon request. PCR was performed using standard conditions.

Prior to whole genome single nucleotide polymorphism (SNP) mapping all families were pre-screened for mutations in *GJB2* by Sanger sequence analysis. For the *MYO15A* locus, microsatellite markers (D17S1843, D17S2196, D17S783 and D17S1824) were genotyped and in families in which haplotype analysis showed compatibility with genetic linkage, Sanger sequence analysis was performed for *MYO15A*. For family 11DF, microsatellite markers (D9S166, D9S1806 and D9S1876) were used for exclusion of the *TMC1* region.

Families with no identified mutation in *GJB2* and *MYO15A* were further genotyped using the Illumina HumanOmniExpress whole genome single nucleotide polymorphism (SNP) microarray (>700 K SNPs) or the Illumina Human Linkage-12 panel according to the manufacturer's protocols. Homozygosity mapping was performed using an online tool Homozygosity Mapper (URL: http://www.homozygositymapper.org; [15]). The logarithm of odds (LOD) score for the homozygous regions identified in family DFR18 was calculated using the Gene Hunter v2.1r5 program in the easyLINKAGE plus v5.08 software package [16]. The exons and exon-intron boundaries of the known candidate genes (*BSND* (NM_057176.2), *GJB2* (NM_004004.5), *HGF* (NM_000601), *MYO15A* (NM_016239.3), *MSRB3* (NM_001031679.2), *SLC26A4* (NM_000441.1), *TMC1* (NM_138691.2), *TMPRSS3* (NM_024022.2) and *TMIE* (NM_147196)) present in the homozygous regions were sequenced in the proband of the families. Segregation analyses for identified mutations in the corresponding families were performed by Sanger sequencing except for family DFR24 and DFR18. In these families, the segregation of *TMPRSS3* and *MSRB3* mutations was analyzed with restriction digestion with the enzymes *AciI* and *TseI*, respectively (New England Biolabs Inc. UK).

Eighty nine ethnically matched controls were sequenced for the novel mutations identified in the current study. Control panel screening of *GJB2* (c.598G>A); *MYO15A* (c.9948G>A), (c.3866+1G>A), (c.8767C>T), (c.8222 T>C); *TMC1* (c.362+18A>G) and *BSND* (c.97G>C), variants was done by sanger sequencing. While for *TMPRSS3* (c.726C>G) and *MSRB3* (c.20T>G) variants *AciI* and *TseI* PCR-RFLP were performed, respectively.

### *In silico* prediction of the identified variants

*In silico* prediction of the identified variants was performed using online prediction tools. Sorting intolerant from tolerant (SIFT: http://sift.jcvi.org; [17]) and Polymorphism Phenotyping v2 (PolyPhen-2: http://genetics.bwh.harvard.edu/pph2; [18]) were used for analyses of missense changes. Effects on splicing were evaluated with NetGene2 (http://www.cbs.dtu.dk/services/NetGene2/; [19]). SignalP 4.0 (http://www.cbs.dtu.dk/services/SignalP-4.0/) was employed to predict the presence and location of the signal peptide/non-signal peptide cleavage sites [20]. In addition, the online tool, Have your Protein Explained (HOPE) (http://www.cmbi.ru.nl/hope/input) was used to predict the three dimensional structural changes at the protein level [21]. The exome variant server (EVS) and an in-house exome database (Human Genetics, Radboud University Medical Centre) were also searched for the presence of putative pathogenic variants.

### Minigene Construction and Splicing Assay (for c.362+18A>G mutation in *TMC1* and c.9948G>A mutation in *MYO15A*)

A plasmid containing the genomic region encompassing exons 3–5 of *RHO* inserted at the EcoRI/SalI sites in the pCI-NEO vector was used for *in vivo* splicing assays [22]. The plasmid was adapted from the Gateway cloning technology (Life technologies) according to the manufacturer's protocol. PCR amplified fragments of wild-type and mutant *TMC1* exon 8, along with flanking intronic sequences were generated with the following primers, 5′-GGGGACAAGTTTGTACAAAAAAGCAGGCTTCtgggtcctaatgttgactgc-3′ and 5′-GGGGACCACTTTGTACAAGAAAGCTGGGTCggatttagaaaatcaatatcaggg-3′, that contain the attB1 and attB2 sites necessary for Gateway cloning. Similarly, amplified fragments of wild-type and mutant *MYO15A* exon 61 were generated using primers, 5′- GGGGACAAGTTTGTACAAAAAAGCAGGCTTCttccccaggagaaatggag-3′ and 5′- GGGGACCACTTTGTACAAGAAAGCTGGGTCcagcttgctgggaaggac-3′. Recombinant vectors were employed for transfection of HEK293T cells that were incubated at 37°C for 24 hours. RNA was then isolated from the transfected cells and reverse transcribed into cDNA and sequenced. Forward primer 5′-cggaggtcaacaacgagtct-3′ and reverse primer 5′-aggtgtaggggatgggagac-3′ were used to amplify and sequence the amplified cDNA fragments along with flanking *RHO* sequences as present in the vector.

## Results

All families in this study were consanguineous (Table S1) and all patients in these families were diagnosed with severe to profound congenital hearing loss. Causal mutations were identified in all 30 families and the 9 novel mutations identified in the current study were not present in any of the 89 ethnically matched control individuals.

**Figure 1:**
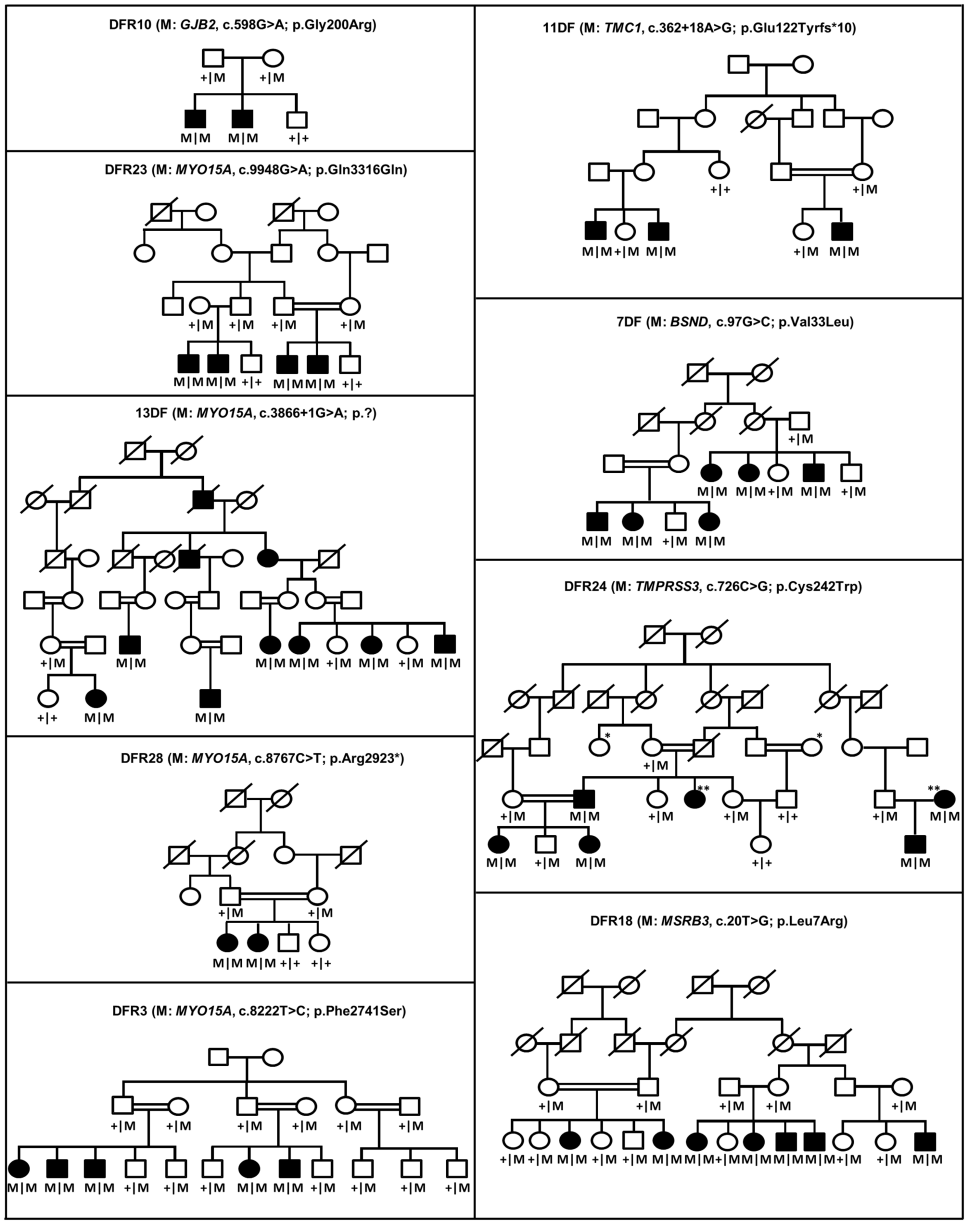
Pedigrees and the segregation of novel mutations in known deafness genes. Unfilled circles indicate unaffected females, unfilled squares indicate unaffected males, filled circles indicate affected females, filled squares indicate affected males, double lines represent consanguineous marriages, slashed line across the symbols indicate deceased individual, + indicates wild type allele, M indicates mutant allele.

Sequence analysis of *GJB2* in the current cohort identified 16 families with mutations in this gene (Table 1; Table 2) segregating with hearing loss. A recurrent nonsense mutation c.71G>A (p.Trp24*) was the most common and was found in 5 families (31%), followed by another nonsense variant, c.231G>A (p.Trp77*) that was identified in 4 families (25%; Table 1). In addition to the 6 recurrent *GJB2* mutations, a novel homozygous change c.598G>A (p.Gly200Arg) was found in one consanguineous family DFR10 (Table 2); as predicted by the HOPE server the larger side chain of the mutant residue arginine might well affect the proper folding of the cysteine rich domain. In addition, the residue resides in the conserved region of the protein (Figure 1; Figure S1A).

**Table 1:** Spectrum of recurrent *GJB2* mutations in Pakistani families with autosomal recessive non-syndromic hearing loss (ARNSHL).

Mutation identified (Protein change)	Type of mutation	No. of families	No. of affected members	Frequency in EVS
c.71G>A (p.Trp24*)	Nonsense (homozygous)	5	26	Absent
c.231G>A (p.Trp77*)	Nonsense (homozygous)	4	14	AA = 0/AG = 1/GG = 4299
c.35delG (p.Gly12Valfs*2)	Frameshift (homozygous)	2	8	Absent
c.35delG (p.Gly12Valfs*2) c.439G>A (p.Glu147Lys)	Missense (compound heterozygous)	1	3	Absent
c.380G>A (p.Arg127His)#	Missense (homozygous)	2	4	AA = 0/AG = 26/GG = 4274
c.377_378insATGCGGA (p.Arg127Cysfs*85)	Frameshift (homozygous)	1	2	Absent

As reference sequence NM_004004.5 was employed. EVS, exome variant server;

# The pathogenicity of this mutation is controversial.

**Table 2:** Novel mutations identified in known genes for autosomal recessive non-syndromic hearing loss (ARNSHL) in the current study.

Family ID	Size of homozygous regions (Mb)	Chr.	Flanking SNPs	Chr. position (in hg19)	Candidate gene (Acc. No.)	Mutation (Predicted protein change)	PhyloP; SIFT; Polyphen	NetGene2	Frequency in EVS
DFR10	ND	13	ND	ND	*GJB2* (NM_004004.5)	Ex-2: c.598G>A (p.Gly200Arg)	3.43; Deleterious; Damaging	NA	Absent
DFR23	ND	17	ND	ND	*MYO15A* (NM_016239.3)	Ex-61: c.9948G>A (p.Gln3316Gln)	NA	Abolition of splice site	Absent
13DF	ND	17	ND	ND	*MYO15A* (NM_016239.3)	In-5: c.3866+1G>A (p.?)	NA	Abolition of splice site	AA = 0, AG = 1, GG = 6051
DFR28	ND	17	ND	ND	*MYO15A* (NM_016239.3)	Ex-50: c.8767C>T (p.Arg2923*)	NA	NA	TT = 0, TC = 1, CC = 6266
DFR3	ND	17	ND	ND	*MYO15A* (NM_016239.3)	Ex-45: c.8222T>C (p.Phe2741Ser)	4.97; Deleterious; Damaging	NA	Absent
11DF	ND	9	ND	ND	*TMC1* (NM_138691.2)	In-8: c.362+18A>G (p.Glu122Tyrfs*10)	NA	NA	Absent
7DF	8.40	1	rs1242330; rs7521242	53,396,842–61,803,889	*BSND* (NM_057176.2)	Ex-1: c.97G>C (p.Val33Leu)	1.09 Deleterious; Damaging	NA	Absent
DFR24	3.49	21	rs2838063; rs881969	42,929,129–46,421,694	*TMPRSS3* (NM_024022.2)	Ex-8: c.726C>G (p.Cys242Trp)	−0.28 Deleterious; Damaging	NA	Absent
DFR18	2.92	12	rs6581511; rs11176432	64,278,102–67,207,064	*MSRB3* (NM_001031679.2)	Ex-4: c.20T>G (p.Leu7Arg)	3.76; Tolerated; Possibly Damaging	NA	Absent

Acc. No., accession number of reference sequence; Chr, chromosome; Ex, exon; EVS, exome variant server; hg19, human genome assembly 19; In, intron; NA, not applicable; ND, not determined; SNPs, single nucleotide polymorphisms; PhyloP, phylogenetic P-values; Polyphen, polymorphism phenotyping; SIFT, sorting intolerance from tolerance.

**Figure 2:**
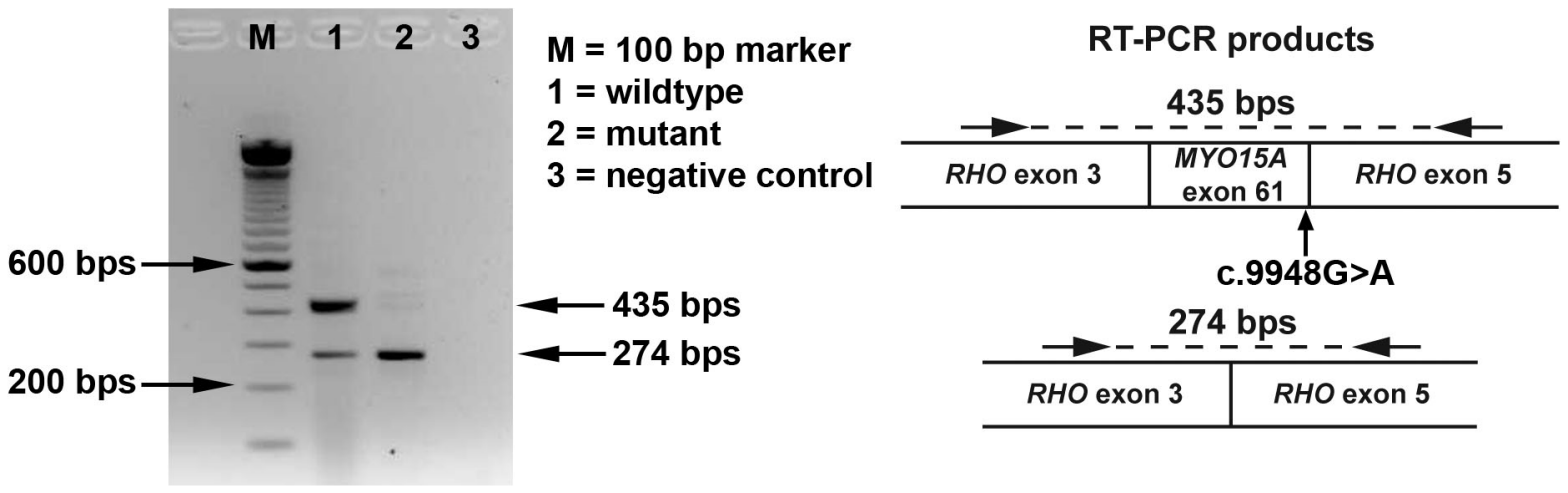
Effect of MYO15A c. 9948G>A using a minigene approach. An agarose gel containing RT-PCR products detected from HEK293T cells transfected with the wildtype and mutant minigene construct and a schematic representation of the identified splicing products. The RT-PCR products were verified by sequence analysis. The c.9948G>A mutation leads to skipping of exon 61.

Out of the 9 novel mutations (Table 2) identified in the current study, 4 were present in *MYO15A*, of which 2 were splice site mutations (c.9948G>A and c.3866+1G>A), one was a nonsense (c.8767C>T) and one a missense mutation (c.8222 T>C). The c.9948G>A variant changes the last nucleotide of exon 61 and is predicted to affect the splice donor site. The splice donor site in the reference sequence has a highly confident score of 94%, which is reduced to a score of 24% in the mutant. To determine the effect of the c.9948G>A mutation on splicing, a minigene approach was used. This showed correct splicing of the wildtype *MYO15A* exon 61, while the c.9948G>A mutation almost completely abolished the normal splicing (Figure 2). NetGene2 predicted the abolition of the splice donor site of exon 5 as a result of the c.3866+1G>A mutation in intron 5 of *MYO15A*. The nonsense mutation c.8767C>T (p.Arg2923*) was novel and predicted to lead to the synthesis of a truncated protein, while the missense mutation c.8222 T>C, leads to the substitution of serine for phenylalanine (p.Phe2741Ser) at amino acid position 2741 that resides in the conserved region of the protein (Figure 1; Figure S1B). This missense change was predicted to be deleterious by SIFT and Polyphen2 (Table 2).

**Figure 3:**
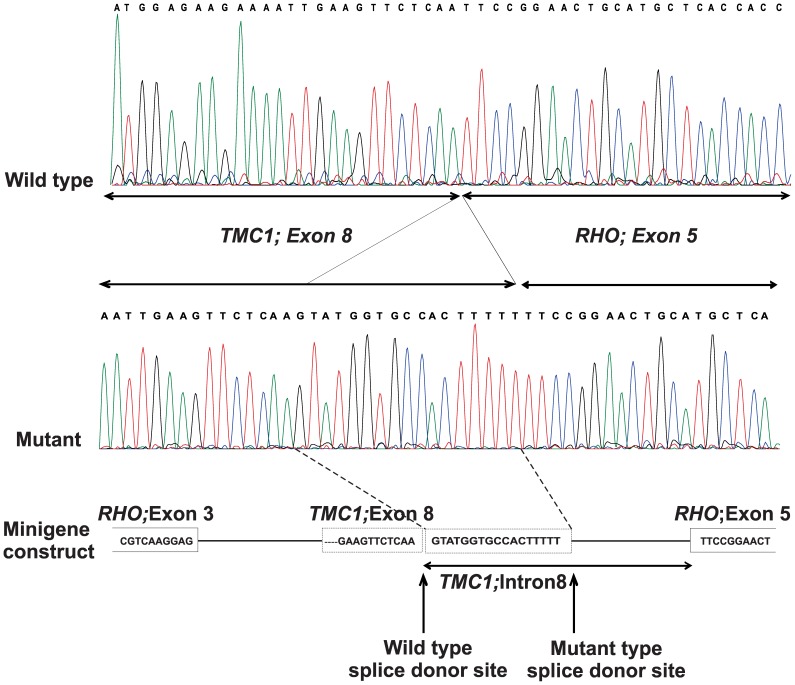
Effect of *TMC1* intronic mutation c.362+18A>G using a minigene approach. Electropherogram of the partial cDNA sequence of RNA derived from cells transfected with the pCI-NEO with either the mutant or wildtype *TMC1* exon 8. The mutation leads to the insertion of 17bp at the 3′end of exon 8, which can be predicted to result in a premature stop codon in exon 9 (p.Glu122Tyrfs*10).

Haplotype analysis of STR-markers flanking *TMC1* showed compatibility with genetic linkage for family 11DF. Since sequence analysis of *TMC1* revealed a novel intronic mutation, c.362+18A>G, this family was not further analyzed by SNP-array genotyping. The variant segregated with the hearing loss in the family (Figure 1) and was predicted to create a novel splice donor site with a similar confidence score as the original splice donor site. To determine the effect of the c.362+18A>G mutation on splicing, a minigene approach was used. This revealed correct splicing of the wild-type *TMC1* exon 8, while the c.362+18A>G mutation resulted in a 17 bp extension of exon 8 (Figure 3). This leads to a frameshift and a premature stop codon (p.Glu122Tyrfs*10).

**Figure 4:**
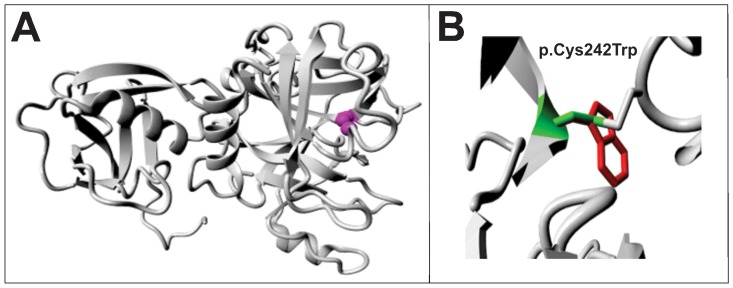
Predicted effect of mutation c.726C>G (p.Cys242Trp) on the three dimensional structure of TMPRSS3. A) Wild type protein structure with an intact disulphide bridge showing position of the mutated residue (magenta). B) Close-up view of the structure showing the wild type residue cysteine (green) and the mutant residue tryptophan (red). In case of the mutant residue there will be no disulphide bridge at this position.

A novel homozygous missense mutation was identified in *TMPRSS3* (MIM_605511), c.726C>G (p.Cys242Trp) that co-segregated with hearing impairment in family DFR24 (Figure 1) and was predicted to be probably damaging by Polyphen2 and deleterious by SIFT (Table 2). This mutation is located in the peptidase S1 domain and the HOPE server predicted the abolition of the catalytic activity of TMPRSS3. In addition the amino acid substitution is present in a region of the protein that is conserved across different species (Figure S1D) and therefore probably affects the core structure of the peptidase domain (Figure 4).

In family DFR18 SNP microarray data analysis revealed a 17.6 Mb homozygous region on chromosome 12 flanked by SNPs rs7978381 and rs7976686, a LOD score of 2.7 was calculated for this region. The known deafness gene, *MSRB3* (MIM_613719), in the region was subsequently sequenced, revealing a novel homozygous nucleotide substitution c.20T>G (p.Leu7Arg) in exon 4 (Figure 1; Table 2). The leucine at position 7 is located in the signal peptide of the MSRB3 protein and as a result of the substitution by arginine this signal peptide loses its function as predicted by SignalP 4.0. Furthermore, the Leu7 residue is conserved across species (Figure S1E). The mutation abolishes a *TseI* restriction site, which allowed the segregation of this variant in the family to be checked by restriction digestion. By using the same analysis, this variant was also found heterozygously in 3 out of 178 ethnically matched control alleles.

The novel *BSND* (MIM_606412) missense mutation c.97G>C (p.Val33Leu) in family 7DF (Figure 1) was predicted to be deleterious by SIFT and Polyphen2 (Table 2) and Val33 is conserved across species (Figure S1C).

Recurrent mutations in *TMC1* (c.1114G>A; p.Val372Met [23] and c.100C>T; p.Arg34* [24]), *HGF* (c.482+1991_2000delGATGATGAAA and c.482+1986_1988delTGA [25]), *SLC26A4* (c.1337A>G; p.Gln446Arg [26]) and *TMIE* (c.241C>T; p.Arg81Cys [27]) also segregated in the respective families and most likely are the disease causing mutations (Table 3).

**Table 3:** Recurrent mutations in known autosomal recessive non-syndromic hearing loss (ARNSHL) genes in 6 Pakistani families.

Family ID	Size of Homozygous Region (Mb)	Chr.	Flanking SNPs	Chr. Position (In hg19)	Candidate Gene	Mutation (Predicted protein change)	Frequency in EVS	Ref.
DFR22	9.23	9	rs4275319; rs2295861	68,513,625–77,745,424	*TMC1*, (NM_138691.2)	Ex-15: c.1114G>A (p.Val372Met)	AA = 0, AG = 2, GG = 6501	[23]
19DFS	11.96	9	rs10867845;rs10867778	72252269–84222230	*TMC1*, (NM_138691.2)	Ex-7: c.100C>T, (p.Arg34*)	Absent	[40]
DFR20	26.06	7	rs10485886; rs2073791	78,057,840–104,122,989	*HGF*, (NM_000601)	In-4: c.482+1991_2000delGATGATGAAA (p.?)	Absent	[25]
DFR37	37.85	7	rs13234819; rs104869	44,342,969–82,197,469	*HGF*, (NM_000601)	In-4: c.482+1986_1988delTGA (p.?)	Absent	[25]
DFR39	29.83	7	rs2285507; rs10253693	92,989,228–122,825,956	*SLC26A4*, (NM_000441.1)	Ex-11: c.1337A>G (p.Gln446Arg)	Absent	[26]
26DF	2652	3	rs304838; rs536036	30806674–57326883	*TMIE* (NM_147196)	Ex-3: c.241C>T (p.Arg81Cys)	Absent	[27]

Acc. No., accession number of reference sequence; Chr, chromosome; Ex, exon; EVS, exome variant server; In, intron; SNPs, single nucleotide polymorphisms; Ref, references.

## Discussion

In a cohort of 30 ARNSHL families the genetic defects were identified in 9 known deafness genes; *GJB2*, *MYO15A*, *TMC1*, *BSND*, *TMPRSS3*, *MSRB3*, *HGF*, *SLC26A4* and *TMIE*. In the current panel recurrent as well as novel mutations were detected, the novel mutations were identified in *GJB2*, *MYO15A*, *TMC1*, *BSND*, *TMPRSS3* and *MSRB3*.

*GJB2* was the most frequently mutated gene in these families. In this gene the novel mutation c.598G>A (p.Gly200Arg) affects a residue in the cysteine-rich domain that is involved in the formation of intramolecular disulphide bonds [28], [29]. Although the effect of glycine on the intramolecular disulphide bond formation cannot be predicted, the mutant residue arginine may affect the proper folding of the cysteine-rich domain because of the larger size of the arginine side chain.

In two families the *GJB2* variant c.380G>A (p.Arg127His) was found to segregate with the hearing loss, being present homozygously in 4 affected members. Other studies have also reported deafness patients carrying this mutation homozygously [29], [30], [31]. Based upon the higher carrier frequency in patients as well as controls, Padma et al. [30] suggested that it is unlikely that this variant plays a pathogenic role in deafness. However, Matos et al. [32] have proposed that this mutation is likely to be pathogenic when influenced by other unknown genetic or environmental factors [32]. In the EVS, however, no homozygous occurrence of the mutated allele has been reported to date (Table S1). Identification of 4 p.Arg127His homozygous patients out of 125 deafness patients in the current study and the previously reported cases of 9 heterozygous cases and 1 compound heterozygous case in 70 deaf patients by Bukhari et al. [31], demonstrates that this is the most frequently occurring mutation in Pakistani deafness families and therefore becomes important in genetic counseling of Pakistani deaf patients.

In the current study *GJB2* mutations were found to be the most common, followed by *MYO15A* mutations. The overall frequency of the two most common nonsense variants c.71G>A (p.Trp24*) and c.231G>A (p.Trp77*) of *GJB2* in Pakistani deafness patients was obtained from the current (n = 125) as well as previously reported studies of Santos et al. [33] (n = 430) and Bukhari et al. [31] (n = 70). Using these data, the frequency was found to be 5.9% (p.Trp24*) and 4.3% (p.Trp77*) [31], [33]. The variant p.Trp24* has a high prevalence in the Indian population as well [34], Pakistan and India have a shared genetic ancestry [35], which could be the reason of the presence of this variant in both populations. The third mutation p.Gly12Valfs*2 identified in the current study has previously been reported from Northern areas of Pakistan [31] and also in Caucasians and Turks [36], thus indicating a possible founder effect of this mutation. In the current cohort, 35% (60/173) individuals were found to be carriers of *GJB2* mutations. Therefore, as a preliminary screening of deaf families the sequencing of this gene is suggested in Pakistan. In the current study *MYO15A* was found to be the second leading cause of deafness in the Pakistani population, but still no recurrent mutation was identified in this gene.

A total of 7 nonsense mutations were identified in *GJB2*, *MYO15A* and *TMC1*, these nonsense mutations are likely to cause nonsense mediated decay (NMD) because they are either present in the middle or near the 5′end of the gene.

In *MYO15A* two splice site, a nonsense and a missense mutations were identified. The c.9948G>A (p.Gln3316Gln) mutation affects the last nucleotide of exon 61 and changes the consensus splice site sequence. Based on the results of the minigene assay the c.9948G>A mutation is expected to lead to skipping of exon 61, which would result in a frameshift and NMD can be expected to occur at least for part of the mRNAs. The second splice site mutation c.3866+1G>A is a canonical splice site change and is predicted to remove the splice donor site of exon 5. An alternative splice site is predicted at position +99 in intron 5, if this alternative site is used it would lead to a frameshift and a premature stop codon (encoded by nucleotide 2–4 of intron 5). Both splice site mutations are thus predicted to lead to a frameshift and NMD could occur for at least part of the mRNA transcripts. The two other novel mutations, c.8222T>C (p.Phe2741Ser) and c.8767C>T (p.Arg2923*), found in two different families, are located in the region of the gene that encodes the tail region of *MYO15A* and are likely to cause a loss of function of this region. The p.Arg2923* mutation is present in the SH3 domain, which is involved in the protein-protein interactions [37]. Most of the previously reported mutations of *MYO15A* causing congenital severe to profound deafness were found in the motor head and the tail domains [6], [38]. Collectively these results indicate that the motor head and tail regions of *MYO15A* are essential in the hearing process and any mutation in these regions is thus critical [39].

*TMC1* encoding a transmembrane protein is expressed in the neurosensory hair cells of the mouse cochlea [40]. The recurrent nonsense mutation in exon 7 of *TMC1*, c.100C>T (p.Arg34*) is the most common *TMC1* mutation reported in Pakistan. It leads to a truncated protein and leads to congenital severe to profound hearing loss [40], [41], [42], [43]. Ben Said et al. [42] have previously described that the p.Arg34* is an old founder mutation found in several populations including the Pakistani population. It is likely that the family described here carries the mutation on the founder haplotype. Also a novel intronic mutation in the *TMC1* gene, c.362+18A>G was identified in the current study (Table 2), which creates a novel splice donor site and the insertion of 17 nucleotides as demonstrated in the minigene approach.

*BSND* encodes the barttin protein, which is a vital subunit of the chloride and voltage-sensitive Ka (CLCNKA) and Kb (CLCNKB) channels in the inner ear and the kidney. Chloride channels and the barttin protein form heteromers which function in the recycling of the K^+^ ions in the inner ear and salt reabsorption in the kidneys [44], [45], [46], [47], [48], [49]. As predicted by *in silico* analysis, the currently identified *BSND* mutation p.Val33Leu residing in the transmembranal region might result in the malfunctioning of chloride channels CLCNKA and CLCNKB. However, in the current family there were no complaints of any renal problems.

Deafness caused by mutations in the *TMPRSS3* gene is bilateral and severe to profound with no defects of the middle ear and the vestibular system [50]. Mutations in *TMPRSS3* can also cause progressive hearing loss with a postlingual onset [51]. *TMPRSS3* mutation c.726C>G (p.Cys242Trp) in exon 8 affects the serine protease domain as predicted by 3D modeling. Previously another mutation in the same exon of *TMPRSS3*, c. 647G>T (p.Arg216Leu) has been shown to result in a failure of the protein to undergo proteolytic cleavage resulting in the inactivation of the sodium channel [52].

The homozygous variant p.Leu7Arg (c.20T>G) in *MSRB3* identified in the current study is located in the mitochondrial signal sequence and may result in mislocalization of the protein. Ahmed et al. [53] have shown the importance of the mitochondrial isoforms when they found a mutation c.55T>C (p.Arg19*) to underlie hearing impairment. This mutation also resides in the signal sequence for mitochondrial localization. However, the variant c.20T>G (p.Leu7Arg) was also found in heterozygous state in 3 out of 89 (1.7%) ethnically matched controls. The mutation might be a founder mutation in the corresponding population. However, currently it is uncertain whether this variant is the cause of hearing impairment in the family and further studies are necessary for a definite conclusion on the pathogenic effect of the variant.

## Conclusions

In the present study 53% (16/30) of the families were found to carry causative mutations in *GJB2* illustrating that the most frequently involved gene in deafness in the Pakistani population is *GJB2* followed by *MYO15A* (13%, 4/30) and *TMC1* (10%, 3/30). Based on these results it is therefore suggested that as an initial step for the genetic diagnosis of deafness, *GJB2* should be analyzed in Pakistani patients and if this gene is excluded then microsatellite markers flanking *MYO15A* and *TMC1* should be genotyped to exclude those genes or to indicate mutation analysis. Homozygosity mapping is an effective approach to determine the mutated genes in consanguineous families. Although a large number of deafness genes and mutations have already been identified, our study demonstrates that the full mutation spectrum in these genes is still not defined. Identification of novel mutations in these genes is important for genetic counseling and can provide handles for further studies on protein function. Genetic counseling of the families is important to better inform couples about the risk of their offspring to be hearing impaired. In addition, as can be seen in the current study, recurrent as well as novel mutations in known genes define the disease in the families which seem to have some population specificity. This information can be important in developing screening strategies.

## Supporting Information

Figure S1 **Multiple-alignment of the corresponding stretches of protein sequences across different species.** The blue color shading represents the intensity of conservation, where the dark blue shading represents highly conserved stretch while the light blue shading denotes moderate conservation of residues across different species. A) Amino acid sequence conservation of p.Gly200 across 9 species. B) Amino acid sequence conservation of p.Phe2741 across 11 species. C) Amino acid sequence conservation of p.Val33 across 7 species. D) Amino acid sequence conservation of p.Cys24 across 10 species. E) Non-conserved residue of p.Leu7 across 10 species.
(TIF)

Table S1 **Characteristics of 30 Pakistani families diagnosed with autosomal recessive non-syndromic hearing loss (ARNSHL).**
(DOC)

## References

[1] Mustapha M, Salem N, Delague V, Chouery E, Ghassibeh M, et al (2001) Autosomal recessive non-syndromic hearing loss in the Lebanese population: prevalence of the 30delG mutation and report of two novel mutations in the connexin 26 (GJB2) gene. J Med Genet 38: E36. 11584050 10.1136/jmg.38.10.e36 PMC1734738

[2] Van Camp G, Coucke PJ, Kunst H, Schatteman I, Van Velzen D, et al (1997) Linkage analysis of progressive hearing loss in five extended families maps the DFNA2 gene to a 1.25-Mb region on chromosome 1p. Genomics 41: 70–74. 9126484 10.1006/geno.1997.4624

[3] Morton NE (1991) Genetic epidemiology of hearing impairment. Ann N Y Acad Sci 630: 16–31. 1952587 10.1111/j.1749-6632.1991.tb19572.x

[4] Bayazit YA, Yilmaz M (2006) An overview of hereditary hearing loss. ORL J Otorhinolaryngol Relat Spec 68: 57–63. 16428895 10.1159/000091090

[5] Elahi MM, Elahi F, Elahi A, Elahi SB (1998) Paediatric hearing loss in rural Pakistan. J Otolaryngol 27: 348–353. 9857321

[6] Hilgert N, Smith RJ, Van Camp G (2009) Forty-six genes causing nonsyndromic hearing impairment: which ones should be analyzed in DNA diagnostics? Mutat Res 681: 189–196. 18804553 10.1016/j.mrrev.2008.08.002 PMC2847850

[7] Zelante L, Gasparini P, Estivill X, Melchionda S, D'Agruma L, et al (1997) Connexin26 mutations associated with the most common form of non-syndromic neurosensory autosomal recessive deafness (DFNB1) in Mediterraneans. Hum Mol Genet 6: 1605–1609. 9285800 10.1093/hmg/6.9.1605

[8] Estivill X, Fortina P, Surrey S, Rabionet R, Melchionda S, et al (1998) Connexin-26 mutations in sporadic and inherited sensorineural deafness. Lancet 351: 394–398. 9482292 10.1016/S0140-6736(97)11124-2

[9] Kelley PM, Harris DJ, Comer BC, Askew JW, Fowler T, et al (1998) Novel mutations in the connexin 26 gene (GJB2) that cause autosomal recessive (DFNB1) hearing loss. Am J Hum Genet 62: 792–799. 9529365 10.1086/301807 PMC1377046

[10] Bashir R, Fatima A, Naz S (2012) Prioritized sequencing of the second exon of MYO15A reveals a new mutation segregating in a Pakistani family with moderate to severe hearing loss. Eur J Med Genet 55: 99–102. 22245518 10.1016/j.ejmg.2011.12.003 PMC3534775

[11] Sambrook J, Russell DW (2006) The condensed protocols from Molecular cloning: a laboratory manual. Cold Spring Harbor, N.Y.: Cold Spring Harbor Laboratory Press v, 800 p.p.

[12] de Heer AM, Collin RWJ, Huygen PL, Schraders M, Oostrik J, et al (2011) Progressive sensorineural hearing loss and normal vestibular function in a Dutch DFNB7/11 family with a novel mutation in TMC1. Audiol Neurootol 16: 93–105. 21252500 10.1159/000313282

[13] Schraders M, Lee K, Oostrik J, Huygen PL, Ali G, et al (2010) Homozygosity mapping reveals mutations of GRXCR1 as a cause of autosomal-recessive nonsyndromic hearing impairment. Am J Hum Genet 86: 138–147. 20137778 10.1016/j.ajhg.2009.12.017 PMC2820176

[14] Rozen S, Skaletsky H (2000) Primer3 on the WWW for general users and for biologist programmers. Methods Mol Biol 132: 365–386. 10547847 10.1385/1-59259-192-2:365

[15] Seelow D, Schuelke M, Hildebrandt F, Nurnberg P (2009) HomozygosityMapper—an interactive approach to homozygosity mapping. Nucleic Acids Res 37: W593–599. 19465395 10.1093/nar/gkp369 PMC2703915

[16] Hoffmann K, Lindner TH (2005) easyLINKAGE-Plus—automated linkage analyses using large-scale SNP data. Bioinformatics 21: 3565–3567. 16014370 10.1093/bioinformatics/bti571

[17] Sim NL, Kumar P, Hu J, Henikoff S, Schneider G, et al (2012) SIFT web server: predicting effects of amino acid substitutions on proteins. Nucleic Acids Res 40: W452–457. 22689647 10.1093/nar/gks539 PMC3394338

[18] Adzhubei IA, Schmidt S, Peshkin L, Ramensky VE, Gerasimova A, et al (2010) A method and server for predicting damaging missense mutations. Nat Methods 7: 248–249. 20354512 10.1038/nmeth0410-248 PMC2855889

[19] Brunak S, Engelbrecht J, Knudsen S (1991) Prediction of human mRNA donor and acceptor sites from the DNA sequence. J Mol Biol 220: 49–65. 2067018 10.1016/0022-2836(91)90380-o

[20] Petersen TN, Brunak S, von Heijne G, Nielsen H (2011) SignalP 4.0: discriminating signal peptides from transmembrane regions. Nat Methods 8: 785–786. 21959131 10.1038/nmeth.1701

[21] Venselaar H, Te Beek TA, Kuipers RK, Hekkelman ML, Vriend G (2010) Protein structure analysis of mutations causing inheritable diseases. An e-Science approach with life scientist friendly interfaces. BMC Bioinformatics 11: 548. 21059217 10.1186/1471-2105-11-548 PMC2992548

[22] Gamundi MJ, Hernan I, Muntanyola M, Maseras M, Lopez-Romero P, et al (2008) Transcriptional expression of cis-acting and trans-acting splicing mutations cause autosomal dominant retinitis pigmentosa. Hum Mutat 29: 869–878. 18412284 10.1002/humu.20747

[23] Santos RL, Wajid M, Khan MN, McArthur N, Pham TL, et al (2005) Novel sequence variants in the TMC1 gene in Pakistani families with autosomal recessive hearing impairment. Hum Mutat 26: 396. 16134132 10.1002/humu.9374 PMC2909098

[24] Searle C, Mavrogiannis LA, Bennett CP, Charlton RS (2012) The common TMC1 mutation c.100C>T (p.Arg34X) is not a significant cause of deafness in British Asians. Genet Test Mol Biomarkers 16: 453–455. 22288896 10.1089/gtmb.2011.0254

[25] Schultz JM, Khan SN, Ahmed ZM, Riazuddin S, Waryah AM, et al (2009) Noncoding mutations of HGF are associated with nonsyndromic hearing loss, DFNB39. Am J Hum Genet 85: 25–39. 19576567 10.1016/j.ajhg.2009.06.003 PMC2706959

[26] Reardon W, O'Mahoney CF, Trembath R, Jan H, Phelps PD (2000) Enlarged vestibular aqueduct: a radiological marker of pendred syndrome, and mutation of the PDS gene. QJM 93: 99–104. 10700480 10.1093/qjmed/93.2.99

[27] Naz S, Giguere CM, Kohrman DC, Mitchem KL, Riazuddin S, et al (2002) Mutations in a novel gene, TMIE, are associated with hearing loss linked to the DFNB6 locus. Am J Hum Genet 71: 632–636. 12145746 10.1086/342193 PMC379198

[28] Batissoco AC, Auricchio MT, Kimura L, Tabith-Junior A, Mingroni-Netto RC (2009) A novel missense mutation p.L76P in the GJB2 gene causing nonsyndromic recessive deafness in a Brazilian family. Braz J Med Biol Res 42: 168–171. 19274344 10.1590/s0100-879x2009000200004

[29] Minarik G, Ferak V, Ferakova E, Ficek A, Polakova H, et al (2003) High frequency of GJB2 mutation W24X among Slovak Romany (Gypsy) patients with non-syndromic hearing loss (NSHL). Gen Physiol Biophys 22: 549–556. 15113126

[30] Padma G, Ramchander PV, Nandur UV, Padma T (2009) GJB2 and GJB6 gene mutations found in Indian probands with congenital hearing impairment. J Genet 88: 267–272. 20086291 10.1007/s12041-009-0039-5

[31] Bukhari I, Mujtaba G, Naz S (2013) Contribution of GJB2 mutations to hearing loss in the Hazara Division of Pakistan. Biochem Genet 51: 524–529. 23504403 10.1007/s10528-013-9583-z PMC3708968

[32] Matos TD, Simoes-Teixeira H, Caria H, Rosa H, O'Neill A, et al (2010) The controversial p.Arg127His mutation in GJB2: report on three Portuguese hearing loss family cases. Genet Test Mol Biomarkers 14: 141–144. 19929408 10.1089/gtmb.2009.0103

[33] Santos RL, Wajid M, Pham TL, Hussan J, Ali G, et al (2005) Low prevalence of Connexin 26 (GJB2) variants in Pakistani families with autosomal recessive non-syndromic hearing impairment. Clin Genet 67: 61–68. 15617550 10.1111/j.1399-0004.2005.00379.x PMC2909104

[34] Mani RS, Ganapathy A, Jalvi R, Srikumari Srisailapathy CR, Malhotra V, et al (2009) Functional consequences of novel connexin 26 mutations associated with hereditary hearing loss. Eur J Hum Genet 17: 502–509. 18941476 10.1038/ejhg.2008.179 PMC2986212

[35] Qamar R, Ayub Q, Mohyuddin A, Helgason A, Mazhar K, et al (2002) Y-chromosomal DNA variation in Pakistan. Am J Hum Genet 70: 1107–1124. 11898125 10.1086/339929 PMC447589

[36] Snoeckx RL, Huygen PL, Feldmann D, Marlin S, Denoyelle F, et al (2005) GJB2 mutations and degree of hearing loss: a multicenter study. Am J Hum Genet 77: 945–957. 16380907 10.1086/497996 PMC1285178

[37] Weng Z, Rickles RJ, Feng S, Richard S, Shaw AS, et al (1995) Structure-function analysis of SH3 domains: SH3 binding specificity altered by single amino acid substitutions. Mol Cell Biol 15: 5627–5634. 7565714 10.1128/mcb.15.10.5627 PMC230813

[38] Belyantseva IA, Boger ET, Friedman TB (2003) Myosin XVa localizes to the tips of inner ear sensory cell stereocilia and is essential for staircase formation of the hair bundle. Proc Natl Acad Sci U S A 100: 13958–13963. 14610277 10.1073/pnas.2334417100 PMC283528

[39] Anderson DW, Probst FJ, Belyantseva IA, Fridell RA, Beyer L, et al (2000) The motor and tail regions of myosin XV are critical for normal structure and function of auditory and vestibular hair cells. Hum Mol Genet 9: 1729–1738. 10915760 10.1093/hmg/9.12.1729

[40] Kitajiri SI, McNamara R, Makishima T, Husnain T, Zafar AU, et al (2007) Identities, frequencies and origins of TMC1 mutations causing DFNB7/B11 deafness in Pakistan. Clin Genet 72: 546–550. 17877751 10.1111/j.1399-0004.2007.00895.x

[41] Sirmaci A, Duman D, Ozturkmen-Akay H, Erbek S, Incesulu A, et al (2009) Mutations in TMC1 contribute significantly to nonsyndromic autosomal recessive sensorineural hearing loss: a report of five novel mutations. Int J Pediatr Otorhinolaryngol 73: 699–705. 19187973 10.1016/j.ijporl.2009.01.005

[42] Ben Said M, Hmani-Aifa M, Amar I, Baig SM, Mustapha M, et al (2010) High frequency of the p.R34X mutation in the TMC1 gene associated with nonsyndromic hearing loss is due to founder effects. Genet Test Mol Biomarkers 14: 307–311. 20373850 10.1089/gtmb.2009.0174 PMC2936956

[43] Hilgert N, Alasti F, Dieltjens N, Pawlik B, Wollnik B, et al (2008) Mutation analysis of TMC1 identifies four new mutations and suggests an additional deafness gene at loci DFNA36 and DFNB7/11. Clin Genet 74: 223–232. 18616530 10.1111/j.1399-0004.2008.01053.x PMC4732719

[44] Estevez R, Boettger T, Stein V, Birkenhager R, Otto E, et al (2001) Barttin is a Cl- channel beta-subunit crucial for renal Cl- reabsorption and inner ear K+ secretion. Nature 414: 558–561. 11734858 10.1038/35107099

[45] Hayama A, Rai T, Sasaki S, Uchida S (2003) Molecular mechanisms of Bartter syndrome caused by mutations in the BSND gene. Histochem Cell Biol 119: 485–493. 12761627 10.1007/s00418-003-0535-2

[46] Janssen AG, Scholl U, Domeyer C, Nothmann D, Leinenweber A, et al (2009) Disease-causing dysfunctions of barttin in Bartter syndrome type IV. J Am Soc Nephrol 20: 145–153. 18776122 10.1681/ASN.2008010102 PMC2615720

[47] Kramer BK, Bergler T, Stoelcker B, Waldegger S (2008) Mechanisms of Disease: the kidney-specific chloride channels ClCKA and ClCKB, the Barttin subunit, and their clinical relevance. Nat Clin Pract Nephrol 4: 38–46. 18094726 10.1038/ncpneph0689

[48] Rickheit G, Maier H, Strenzke N, Andreescu CE, De Zeeuw CI, et al (2008) Endocochlear potential depends on Cl- channels: mechanism underlying deafness in Bartter syndrome IV. EMBO J 27: 2907–2917. 18833191 10.1038/emboj.2008.203 PMC2580783

[49] Waldegger S, Jeck N, Barth P, Peters M, Vitzthum H, et al (2002) Barttin increases surface expression and changes current properties of ClC-K channels. Pflugers Arch 444: 411–418. 12111250 10.1007/s00424-002-0819-8

[50] Fasquelle L, Scott HS, Lenoir M, Wang J, Rebillard G, et al (2011) Tmprss3, a transmembrane serine protease deficient in human DFNB8/10 deafness, is critical for cochlear hair cell survival at the onset of hearing. J Biol Chem 286: 17383–17397. 21454591 10.1074/jbc.M110.190652 PMC3089580

[51] Weegerink NJ, Schraders M, Oostrik J, Huygen PL, Strom TM, et al (2011) Genotype-phenotype correlation in DFNB8/10 families with TMPRSS3 mutations. J Assoc Res Otolaryngol 12: 753–766. 21786053 10.1007/s10162-011-0282-3 PMC3214237

[52] Elbracht M, Senderek J, Eggermann T, Thurmer C, Park J, et al (2007) Autosomal recessive postlingual hearing loss (DFNB8): compound heterozygosity for two novel TMPRSS3 mutations in German siblings. J Med Genet 44: e81. 17551081 10.1136/jmg.2007.049122 PMC2752172

[53] Ahmed ZM, Yousaf R, Lee BC, Khan SN, Lee S, et al (2011) Functional null mutations of MSRB3 encoding methionine sulfoxide reductase are associated with human deafness DFNB74. Am J Hum Genet 88: 19–29. 21185009 10.1016/j.ajhg.2010.11.010 PMC3014371

