# Supplementary figures and images for: Genetic Spectrum of Autosomal Recessive Non-Syndromic Hearing Loss in Pakistani Families
Source: PLoS One. 2014 Jun 20;9(6):e100146. doi: 10.1371/journal.pone.0100146 (PMC4065008; doi:10.1371/journal.pone.0100146)

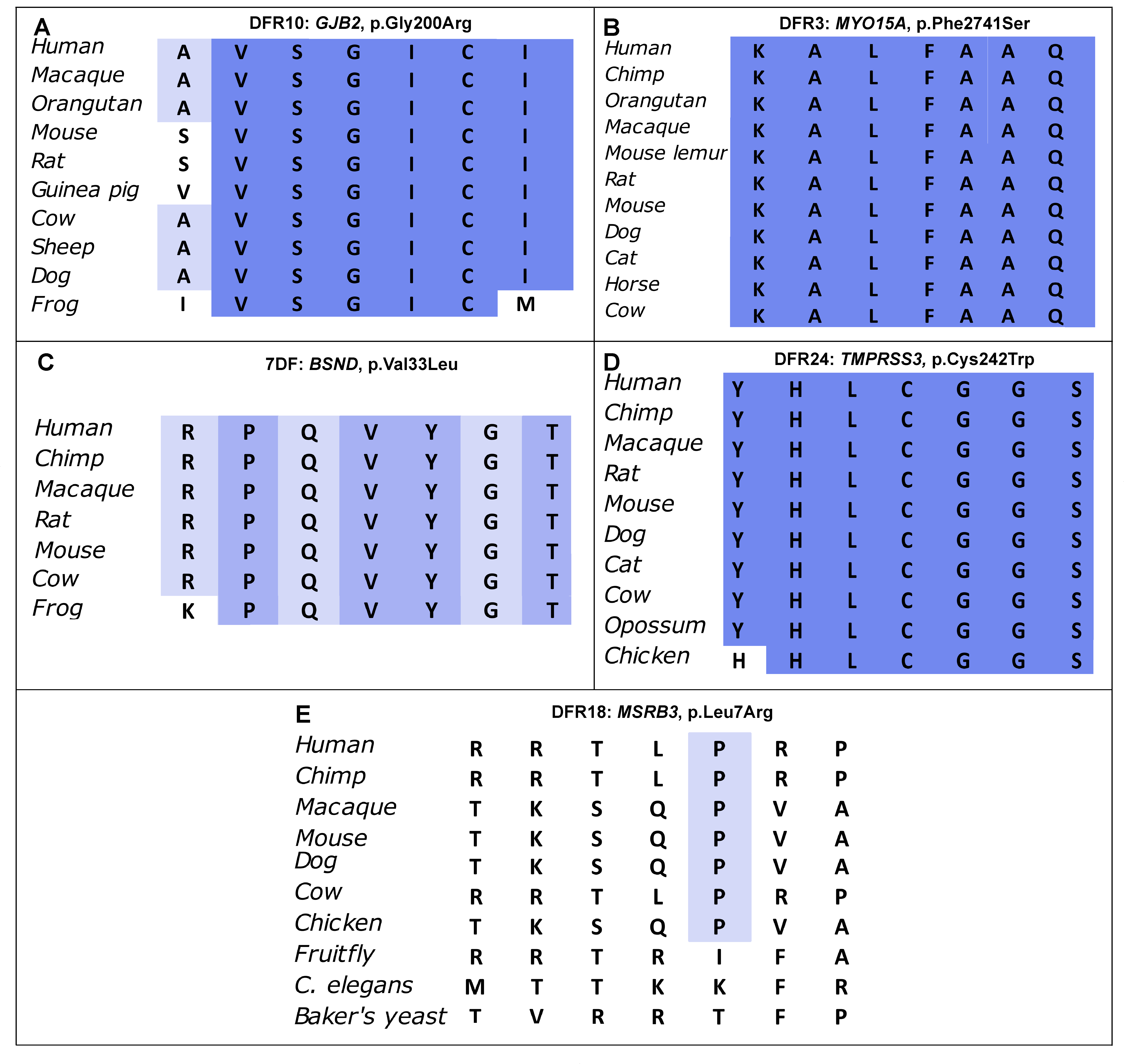

Supplement: Figure S1 — Multiple-alignment of the corresponding stretches of protein sequences across different species. The blue color shading represents the intensity of conservation, where the dark blue shading represents highly conserved stretch while the light blue shading denotes moderate conservation of residues across different species. A) Amino acid sequence conservation of p.Gly200 across 9 species. B) Amino acid sequence conservation of p.Phe2741 across 11 species. C) Amino acid sequence conservation of p.Val33 across 7 species. D) Amino acid sequence conservation of p.Cys24 across 10 species. E) Non-conserved residue of p.Leu7 across 10 species. (TIF) [file pone.0100146.s001.tif]
