# Supplementary material for: Genetic Spectrum of Autosomal Recessive Non-Syndromic Hearing Loss in Pakistani Families
Source: PLoS One. 2014 Jun 20;9(6):e100146. doi: 10.1371/journal.pone.0100146 (PMC4065008; doi:10.1371/journal.pone.0100146)
Supplement: Table S1 — Characteristics of 30 Pakistani families diagnosed with autosomal recessive non-syndromic hearing loss (ARNSHL). (DOC) [file pone.0100146.s002.doc]

**Table S1.** Characteristics of 30 Pakistani families diagnosed with autosomal recessive non-syndromic hearing loss (ARNSHL).

| **Family ID** | **Mutated Gene** | **Identified Mutation** | **Consanguinity** | **Sampled individuals** | **Affected individuals** | **Analysis method** |
| --- | --- | --- | --- | --- | --- | --- |
| DFR3 | *MYO15A* | c.8222T>C (p.Phe2741Ser) | First cousin marriage in different loops | 18 | 5 | VNTR marker analysis |
| DFR10 | *GJB2* | c.598G>A; p.Gly200Arg | Consanguinity | 5 | 2 | Sanger sequencing |
| DFR18 | *MSRB3* | c.20T>G (p.Leu7Arg) | Consanguinity in different loops | 19 | 7 | SNP based array |
| DFR19 | *GJB2* | c.231G>A (p.Trp77*) | Consanguinity | 9 | 5 | Sanger sequencing |
| DFR20 | *HGF* | c.482+1991_2000delGATGATGAAA (p.?) | First cousin marriage | 15 | 3 | SNP based array |
| DFR22 | *TMC1* | c.1114G>A (p.Val372Met) | Consanguinity in different loops | 18 | 8 | SNP based array |
| DFR23 | *MYO15A* | Ex-61: c.9948G>A (p.Gln3316Gln) | First cousin marriage in different loops | 10 | 4 | SNP based array |
| DFR24 | *TMPRSS3* | c.726C>G (p.Cys242Trp) | First cousin marriage in 1^st^ and 2^nd^ generation | 13 | 5 | SNP based array |
| DFR27 | *GJB2* | c.71G>A (p.Trp24*) | First cousin marriage | 20 | 6 | Sanger sequencing |
| DFR28 | *MYO15A* | c.8767C>T (p.Arg2923*) | First cousin marriage | 6 | 2 | VNTR marker analysis |
| DFR33 | *GJB2* | c.71G>A (p.Trp24*) | First cousin marriage | 9 | 3 | Sanger sequencing |
| DFR34 | *GJB2* | c.71G>A (p.Trp24*) | First cousin marriage | 13 | 4 | Sanger sequencing |
| DFR35 | *GJB2* | c.231G>A (p.Trp77*) | No consanguinity reported | 6 | 2 | Sanger sequencing |
| DFR37 | *HGF* | In-4: c.482+1986_1988delTGA (p.?) | First cousin marriage in 3^rd^ generation | 8 | 3 | SNP based array |
| DFR39 | *SLC26A4* | c.1337A>G (p.Gln446Arg) | First cousin marriage | 9 | 3 | SNP based array |
| DFR43 | *GJB2* | c.231G>A (p.Trp77*) | First cousin marriage | 8 | 4 | Sanger sequencing |
| DFR45 | *GJB2* | c.231G>A (p.Trp77*) | First cousin marriage | 12 | 3 | Sanger sequencing |
| 2DF | *GJB2* | c.71G>A (p.Trp24*) | First cousin marriage in 3^rd^ generation | 26 | 7 | Sanger sequencing |
| 7DF | *BSND* | c.97G>C (p.Val33Leu) | First cousin marriage in 2^nd^ generation | 10 | 6 | SNP based array |
| 8DF | *GJB2* | c.35delG (p.Gly12Valfs*2) | First cousin marriage | 13 | 4 | Sanger sequencing |
| 9DF | *GJB2* | c.35delG (p.Gly12Valfs*2) | First cousin marriage in different loops | 12 | 4 | Sanger sequencing |
| 11DF | *TMC1* | c.362+18A>G ( p.Glu122Tyrfs*10) | First cousin marriage in 2^nd^ generation | 7 | 3 | VNTR marker analysis |
| 13DF | *MYO15A* | c.3866+1G>A (p.?) | First cousins marriage in different loops | 11 | 7 | VNTR marker analysis |
| 19DFS | *TMC1* | c.100C>T (p.Arg34*) | First cousin marriage | 14 | 5 | SNP based array |
| 25DFS | *GJB2* | c.35delG (p.Gly12Valfs*2) c.439G>A (p.Glu147Lys) | First cousin marriage in 2^nd^ generation | 5 | 4 | Sanger sequencing |
| 26DF | *TMIE* | c.241C>T (p.Arg81Cys) | First cousin marriage | 18 | 4 | SNP based array |
| 32DFS | *GJB2* | c.71G>A (p.Trp24*) | First cousin marriage in different loops | 18 | 6 | Sanger sequencing |
| 37DFS | *GJB2* | c.377_378insATGCGGA (p.Arg127Cysfs*85) | First cousin marriage in 2^nd^ generation | 4 | 2 | Sanger sequencing |
| 38DFS | *GJB2* | c.380G>A (p.Arg127His)^#^ | First cousin marriage | 9 | 3 | Sanger sequencing |
| 39DFS | *GJB2* | c.380G>A (p.Arg127His)^#^ | First cousin marriage | 4 | 1 | Sanger sequencing |

Note: Disease onset and severity in all families was congenital and severe to profound, respectively.

ID, identification number; SNP, single nucleotide polymorphism; VNTR, variable number of tandem repeats
